# Prevalence and determinants of differences in cystatin C and creatinine-based estimated glomerular filtration rate in community-dwelling older adults: a cross-sectional study

**DOI:** 10.1186/s12882-017-0759-3

**Published:** 2017-12-04

**Authors:** Helen Legrand, Karin Werner, Anders Christensson, Mats Pihlsgård, Sölve Elmståhl

**Affiliations:** 1 Department of Clinical Sciences Malmö, Lund University, Jan Waldenströms gata 35, 20502 Malmö, Sweden; 2 Department of Geriatrics, Skåne University Hospital, Malmö, Sweden; 3 Department of Clinical Sciences Malmö, Lund University, Malmö, Sweden; 4 Department of Nephrology, Skåne University Hospital, Malmö, Sweden; 5 Jan Waldenströms gata 15, plan 5, 205 02 Malmö, Sweden

**Keywords:** Cystatin C, Creatinine, Glomerular filtration rate, Older adults, Community-dwelling

## Abstract

**Background:**

Differences in cystatin C and creatinine-based estimated glomerular filtration rate (eGFR) can lead to clinical uncertainty. Existing eGFR equations perform poorly in a subset of individuals. This study aims to describe the prevalence of differences between cystatin C-based (eGFR_cys_) and creatinine-based (eGFR_creat_) eGFR in older adults and to explore which subsets of individuals may be most affected by differing estimations.

**Methods:**

In this cross-sectional study, participants from a cohort of community-dwelling older adults were examined at a baseline visit in 2001-2004 as part of the larger “Good Aging in Skåne” study. Exposure variables were obtained from questionnaires, interviews, examinations, and medical records. Blood samples were taken during the baseline visit, cryopreserved, and analyzed at a later time for biomarkers. The CKD-EPI equations were used to estimate GFR. Initial descriptive analyses were performed on 2931 individuals. A total of 2532 participants were included in the final multiple linear regression.

**Results:**

Nearly two-thirds of participants had eGFR differences exceeding 10%, with nearly 20 % of participants having eGFR differences exceeding 30%. Smoking, age, body mass index (BMI), C-reactive protein (CRP), glucocorticoid use, and mean eGFR were correlated with differences between eGFR_creat_ and eGFR_cys_.

**Conclusions:**

Differences between eGFR_creat_ and eGFR_cys_ are common and often of large magnitude in this community-dwelling population of older adults. The finding of multiple non-GFR determinants correlated to differences in GFR estimations can help direct future research to improve eGFR equations for subgroups prone to conflicting GFR estimations or to guide choice of biomarker for GFR estimation in these subgroups.

**Electronic supplementary material:**

The online version of this article (10.1186/s12882-017-0759-3) contains supplementary material, which is available to authorized users.

## Background

Glomerular filtration rate (GFR) estimating equations based on creatinine and cystatin C perform well overall but yield different and sometimes contradictory results in a subset of individuals [1–10]. Clinicians are chiefly concerned with the quality of estimated GFR (eGFR) for individual patients for drug dosing, diagnosis of kidney disease, and follow-up of kidney function. To improve GFR estimation methods at the individual level it is first necessary to understand which, if any, particular subgroups are prone to poor estimation using one or both biomarkers. Biomarker concentrations are affected by GFR and non-GFR determinants, some of which are corrected for in current estimation equations, for example with coefficients for race and sex. Better understanding of affected subgroups could lead to recommendations on which biomarker to favor when choosing an equation for a patient in a particular subgroup, or could lead to development of additional correction coefficients for non-GFR determinants of biomarker concentrations. Some recent studies have also indicated that a large difference between cystatin C based and creatinine based eGFR can be a marker for increased risk for morbidity and mortality [11–13]. The pathophysiology of this association has not been determined although glomerular pore size has been hypothesized to play a role. Knowledge of which groups may be more prone to large differences in GFR estimates may give insight into this phenomenon.

Most previous studies have focused on determining which eGFR equation is optimal compared to a gold standard. Several studies have examined factors other than GFR affecting creatinine or cystatin C [14–33]. Few have looked specifically at factors affecting consistency between different eGFR equations [34–38].

The first objective of this study is to determine the prevalence and size of differences in GFR estimation using creatinine-based (eGFR_creat_) or cystatin C-based (eGFR_cys_) equations in a community-dwelling population of older adults. The main objective of the study is to explore correlations between non-GFR determinants of biomarkers and differences in eGFR with the aim of finding subgroups for further study.

## Methods

### Population

We used a cross-sectional analysis of the longitudinal cohort study Good Aging in Skåne (GÅS) to study the prevalence and size of eGFR differences as well as their association with selected non-GFR determinants (exposure variables). GÅS is a longitudinal cohort study with an initial baseline population of 2931 individuals 60 to 93 years of age, recruited from nine age cohorts (60, 66, 72, 78, 81, 84, 87, 90, and 93+) from five rural and urban municipalities in southern Sweden. Selection was at random on the basis of the National population register. Oversampling was used in older age groups for better power. Participants were invited by letter with telephone follow-up, with continuous recruitment to reach a goal of 3000 participants. Eligibility criteria were the ability to speak and understand Swedish and to still be living within the study area at time of recruitment. Baseline study visits began January 8, 2001 and ended July 30, 2004. Visits took place at one of the study clinics or at the place of residence if the participant was unable to come to a study clinic.

### Variables

The outcome variable in both components of the analysis was the difference obtained by substracting eGFR_creat_ from eGFR_cys_, divided by the mean value of the two equation results. The resulting values were positive or negative percentages. The eGFR equations used are from the CKD-EPI collaboration [10]. Although they were not developed in elderly populations, the CKD-EPI equations were chosen because they include both eGFR_creat_ and eGFR_cys_ and have been validated in multiple elderly populations, including in a subset of the current study population [9, 39–42]. Ethnicity was not recorded in this study. Due to the preponderance of participants of European ancestry in this cohort the equations were calculated under the assumption that all participants were of European ancestry.

In the descriptive part of the analysis the outcome variable was collapsed into categories of size of difference in eGFR (<10%, 10–30%, >30%). These categories were chosen based on the commonly-used cut-off points for describing the accuracy of GFR estimating equations [43]. Prevalence of each category was calculated for the categories of age by decades, sex, and mean eGFR based on the average of eGFR_creat_ and eGFR_cys_.

Multiple factors appearing to affect serum concentration of creatinine and cystatin C have previously been reported [14–33, 44–47]. The most well-described of these were included in the second part of the analysis. The exposure variables tested were age, sex, smoking, hypertension, BMI, diabetes, thyroid function, glucocorticoid use, CRP level, and mean eGFR. Data on these variables were collected at baseline visits to the study clinic during examination by a registered nurse and a physician, including consultation of participant medical records, and by participant self-report through interviews and questionnaires. Cystatin C and creatinine were analyzed based on plasma samples taken from participants at the baseline study visit and subsequently cryopreserved to be analyzed as one batch at a later time. Details of variable collection methods can be found in Additional file 1.

### Statistical analysis

For the main part of the analysis multiple linear regression was performed using the General Linear Model procedure in SPSS (Version 22.0. Armonk, NY: IBM Corp.). The exposure variables thyroid status (hypothyroid or hyperthyroid relative to euthyroid), smoking status (current or former smoker relative to non-smoker), sex (male relative to female), hypertension, diabetes, glucocorticoid use, age, BMI, CRP, and mean eGFR (all detailed in Additional file 1) were simultaneously entered into the equation. Cases with missing data points were excluded listwise. Testing of model assumptions was performed with Q-Q plotting and plotting of standardized residuals by predicted values.

## Results

**Fig. 1:**
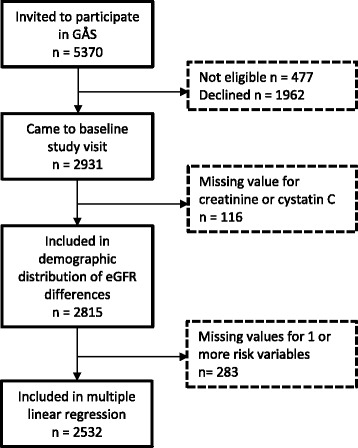
Participant flow

The final multiple linear regression analysis included 2532 individuals. Participant flow is detailed in Fig. 1. Descriptive data on the prevalence of the exposure variables studied is detailed in Table 1.

**Table 1:** Distribution of exposure variables in the study population (*n* = 2931)

**Categorical variables**		
	n (%)	n missing (%)
Thyroid function		38 (1.3)
*Hypothyroid*	171 (5.8)	
*Hyperthyroid*	88 (3.0)	
*Euthyroid*	2634 (90)	
Smoking		114 (3.9)
*Current*	474 (16)	
*Former*	1067 (36)	
*Never*	1276 (44)	
Sex^a^		–
*Male*	1295 (44)	
*Female*	1636 (56)	
Treated for hypertension	849 (29)	26 (0.9)
Diabetes	229 (7.8)	16 (0.5)
Glucocorticoid use^a^	104 (3.5)	–
**Continuous variables**		
	mean (SD)	n missing (%)
Age^a^	73.1 (11)	–
BMI (kg/m^2^)	26.8 (4.4)	80 (2.7)
CRP (mg/L)	5.1 (10)	180 (6.1)
Mean eGFR (mL/min/1.73 m^2^)	66 (19)	116 (4.0)

^a^Variables with theoretically complete data available for all participants

### Main results

Results of the preliminary descriptive study of the prevalence and size of differences in eGFR in this population are presented in Table 2. The mean age of the population was 73.1 years (SD 11) and the mean eGFR was 66 (SD 19). Of note, 526 individuals (19%) had a greater than 30% difference between eGFR_creat_ and eGFR_cys_. The descriptive study also showed a trend towards increasing differences between GFR estimates with increasing age and with decreasing mean eGFR. The mean difference was −1,5 ml/min/1.73m^2^ (SD 15) indicating generally higher eGFR_creat_ than eGFR_cys_.

**Table 2:** Demographic distribution of absolute percent difference in eGFR, 100× (eGFR_cys_ – eGFR_creat_)/mean eGFR (*n* = 2815)

Difference in eGFR	<10%	10-30%	>30%
	n (%)	n (%)	n (%)
Age			
60-69	520 (39)	653 (49)	165 (12)
70-79	220 (40)	240 (45)	79 (15)
80+	249 (27)	407 (43)	282 (30)
Sex			
Male	437 (35)	589 (47)	228 (18)
Female	552 (35)	711 (46)	298 (19)
Mean eGFR (mL/min/1.73 m^2^)			
< 45	106 (26)	165 (40)	140 (34)
45-60	181 (30)	267 (44)	165 (27)
> 60	702 (39)	868 (48)	221 (12)
Total	989 (35)	1300 (46)	526 (19)

The outcome of the multiple linear regression analysis of biomarker determinants on the percent difference in eGFR is detailed with confidence intervals in Table 3. Current smoking (relative to never smoking) and glucocorticoid use were associated with 10 and 9 percentage points greater difference between eGFR values, respectively (former smoking did not differ significantly from never smoking). Each year of increasing age, unit of increased BMI, and mg/L of increased CRP were associated with 1, 0.3 and 0.3 percentage points of greater difference in estimated eGFR, respectively. An increase in mean eGFR was associated with a higher eGFR_cys_, (0,2 percentage points greater difference per mL/min/1.73 m2). Note, however, that this parameter must be interpreted with caution as its statistical significance may well be an artefact of the construction of the outcome. Diabetes, hypo- and hyperthyroidism, sex, and treated hypertension did not seem to affect the difference between eGFR_creat_ and eGFRcys.

**Table 3:** Results of multiple linear regression of biomarker determinants on the percent difference in eGFR

Biomarker determinants	Unstandardized β coefficient	95% Confidence Interval
Hypothyroid	0.16	-3.03, 3.35
Hyperthyroid	-0.79	-5.02, 3.44
Current smoker	-9.88	-12.1, -7.71
Former smoker	-0.29	-1.99, 1.41
Sex	1.36	-0.21, 2.93
Treated for hypertension	0.56	-1.17, 2.29
Diabetes	-0.97	-2.95, 2.76
Glucocorticoid use	-8.78	-12.9, -4.69
Age (years)	-0.76	-0.87, -0.66
BMI (kg/m^2^)	-0.25	-0.42, -0.07
CRP (mg/L)	-0.29	-0.37, -0.21
Mean eGFR (mL/min/1.73 m^2^)	0.22	0.16, 0.28

Percent difference in eGFR expressed as: 100 times the value of (eGFR_cys_ – eGFR_creat_)/mean eGFR (*n* = 2532)

## Discussion

This study demonstrates that in a community-dwelling population of older adults, eGFR_creat_ and eGFR_cys_ yield estimates that differ by more than 10% in nearly two-thirds of cases. In nearly 20 % of cases the two estimates differ by more than 30%. Our results show that smoking, age, BMI, CRP and glucocorticoid use are correlated with increasing eGFR_creat_ or decreased eGFR_cys_ while mean eGFR displays the inverse correlation.

Although it is not possible from this study to know whether the findings are chiefly attributable to effects on creatinine or cystatin C, these findings are not in conflict with previous research on biomarker determinants [15–18, 21–23, 30, 32, 34, 36, 38, 44]. Hypothetical explanations for the correlations seen in this study include a decrease in serum creatinine due to loss of muscle mass in the case of increasing age, prolonged glucocorticoid use or inflammatory disease; the latter being reflected by increased CRP. There have been numerous hypotheses regarding the influence of non-GFR determinants, including body composition, on cystatin C but the physiological mechanisms are not as clear as in the relationship between muscle mass and creatinine levels. Results have often been conflicting regarding the extent of influence of lean body mass, adiposity, diabetes, smoking, level of inflammation, and thyroid function on the cystatin C concentration in blood [16, 19–21, 23, 25, 26, 28, 33, 35–37, 48]. In addition to the potential effect of non-GFR determinants on cystatin C and creatinine production, these biomarkers may also be differentially eliminated in the glomeruli. Previous research has hypothesized the existence of a shrunken pore syndrome, wherein various pathological factors could lead to changes in glomerular membrane pore diameter [11, 49–51]. This could in turn explain differing filtration rates of differently-sized macromolecules, in this case creatinine (113 Da) and cystatin C (13.3 kilodaltons). Recent studies in select adult populations have shown that close to 10% of patients studied display a ratio of eGFR_cys_ to eGFR_creat_ less than or equal to 0.6, which has been defined by researchers as indicative of shrunken pore syndrome [49]. These patients are at generally increased risk for morbidity and mortality, and at higher risk for right ventricular dysfunction and for death after coronary artery bypass grafting [11–13]. It is unknown to what extent the hypothesized shrunken pore syndrome may explain differences in GFR estimates in elderly populations compared to other non-GFR determinants of cystatin C and creatinine that were found to be correlated to differences in eGFR in the current study. The above hypothetical pathophysiological models for the observed differences in GFR estimates can be kept in mind when designing future studies.

The results of this study should prompt clinicians to consider whether one or both biomarkers should be used for GFR estimation in older adults. A clinically important difference between eGFR_cys_ and eGFR_creat_ is not unusual and should be anticipated when the patient profile includes factors known to affect biomarkers.

A strength of this study is its sampling from a general community-dwelling population of elderly both with and without chronic kidney disease.

Choice of risk variables was based on factors known to affect non-GFR determinants of creatinine and cystatin C. One of the strengths of this study is that most factors known to significantly affect biomarkers are included as variables. However, chiefly for reasons of power, not all potential variables were included. For instance, usage of cimetidine or trimethoprim, which have been associated with changes in creatinine metabolism [24, 45, 47], were not included as the total number of study participants taking one of these medications was only six (0.2%).

This study is subject to the common self-selection bias of participants that are healthier on average than the general population of older adults, despite efforts to minimize bias by offering home visits to participants unable to come to the study center. Our assumption is that a healthier population with fewer individuals in the disease-defined biomarker determinant categories decreases the chances of finding correlations between risk variables and the outcome variable. In this age cohort in the study region there are few, if any, non-white individuals. This could affect the generalizability of these results. Race was not recorded during the study, so the exact number of non-white participants is not known.

Although efforts were made to decrease misclassification of exposure variables by accessing both medical records and participant self-report, some of the variables may be subject to bias, most notably variables that rely on medical diagnoses. These are the categories of treatment for hypertension, diabetes, and thyroid function. Hypertension, diabetes, and thyroid function are not routinely screened for in Sweden and therefore it is likely that some proportion of participants were undiagnosed, potentially leading to a misclassification bias. It is also important to mark a distinction between the presence of illness and the presence of treated illness. However, generally speaking, in the Swedish healthcare system all patients with diagnosed diabetes, current imbalances in thyroid hormone production or clinically relevant hypertension receive treatment. The above limitations would tend to bias towards unity and may explain why we were unable to find a correlation between these factors and differences in eGFR_creat_ and eGFR_cys_.

A factor limiting the scope of our study is the lack of measured GFR, meaning we are limited to exploratory analyses of the correlations between biomarker determinants and inconsistencies in estimated GFR. In addition, our consideration of anthropometrics was limited to the use of BMI. Specific consideration of muscle mass and adiposity in relation to the biomarkers and eGFR would require a more accurate measure of body composition, such as Dual-Energy X-ray Absorptiometry (DEXA).

## Conclusions

Our findings suggest that efforts to improve GFR estimating equations may benefit from including the non-GFR determinants smoking, age, BMI, CRP and glucocorticoid use in future analyses to determine if correction coefficients could improve estimation in subpopulations. Some of the affected subpopulations are large, as exemplified by the 16.2% of our population who were current smokers, meaning that the clinical impact of adjustments in eGFR could be substantial. Other future studies could focus on subgroup analysis of risk categories to determine whether one biomarker is superior to the other in particular risk groups.

## Additional file

Additional file 1:
*Variable methods* describing the collection method and coding for each study variable. (PDF 102 kb)

## Abbreviations

BMI: Body mass index
CKD-EPI: Chronic kidney disease epidemiology collaboration
CRP: C-reactive protein
eGFR: Estimated glomerular filtration rate
eGFR_creat_: Creatinine-based eGFR
eGFR_cys_: Cystatin C-based eGFR
GÅS: Good Aging in Skåne
GFR: Glomerular filtration rate
SD: Standard deviation
