# Supplementary material for: Prevalence and determinants of differences in cystatin C and creatinine-based estimated glomerular filtration rate in community-dwelling older adults: a cross-sectional study
Source: BMC Nephrol. 2017 Dec 4;18:350. doi: 10.1186/s12882-017-0759-3 (PMC5716370; doi:10.1186/s12882-017-0759-3)
Supplement: Additional file 1: — Variable methods describing the collection method and coding for each study variable. (PDF 102 kb) [file 12882_2017_759_MOESM1_ESM.pdf]

Variable methods

Variable	Collection method	Coding
Sex	Extracted from personal identity number, which includes coding for sex.	Male or female
Age	Calculated from study visit date and personal identity number, which includes birth date.	Continuous variable
Smoking	Self-reported by participants in a questionnaire; possible answers to the question “do you smoke” were: “yes, regularly,” “yes, occasionally,” “no, no longer smoke,” and “no, have never smoked.”	Regular or occasional smoking classified as current, no longer smoke classified as former, third category never smoked
Hypertension	Medical questionnaire completed by physician based on the medical record and participant report. Question was: “is patient currently under treatment for hypertension?”	Yes or no
BMI	Calculated from current weight and height collected by nurse during nursing portion of the baseline exam.	Continuous variable
Diabetes	Medical questionnaire completed by physician based on the medical record and participant report. One question for diabetes type 1 and one question for diabetes type 2.	Participants with at least one diabetes diagnosis coded as yes, those without any diabetes diagnosis coded as no
Thyroid function	Medical questionnaire completed by physician. First question whether participant has thyroid illness. If yes, follow-up question to categorize whether hypo- or hyperthyroid.	Categories hypothyroid, hyperthyroid, euthyroid
Cystatin C	Blood sample taken at baseline study by nurse, frozen, and analyzed in one batch in 2007 by hospital laboratory using Gentians reagent with a Beckman Coulter LX 20.	Continuous variable
Creatinine	Blood sample taken at baseline study by nurse, frozen, and analyzed in one batch in 2007 by hospital laboratory using a modified Jaffe method with a Beckman Coulter LX 20 traceable to IDMS.	Continuous variable
CRP	Blood sample taken at baseline study by nurse and analyzed daily by hospital laboratory.	Continuous variable
Glucocorticoid use	Medical questionnaire completed by physician based on the medical record and participant report. Question asks for all current medications taken by the patient, both daily and as needed. Glucocorticoids selected by ATC code H02AB.	Yes or no
